# Women’s Experiences with Compliance with Pelvic Floor Home Exercise Therapy and Lifestyle Changes for Pelvic Organ Prolapse Symptoms: A Qualitative Study

**DOI:** 10.3390/jpm12030498

**Published:** 2022-03-19

**Authors:** María Torres-Lacomba, Beatriz Navarro-Brazález, María José Yuste-Sánchez, Beatriz Sánchez-Sánchez, Virginia Prieto-Gómez, Fernando Vergara-Pérez

**Affiliations:** Physiotherapy in Women’s Health (FPSM) Research Group, Physiotherapy Department, Faculty of Medicine and Health Sciences, University of Alcalá, 28805 Madrid, Spain; maria.torres@uah.es (M.T.-L.); marijo.yuste@uah.es (M.J.Y.-S.); beatriz.sanchez@uah.es (B.S.-S.); v.prieto@uah.es (V.P.-G.); fernando.vergara@uah.es (F.V.-P.)

**Keywords:** pelvic organ prolapse, pelvic floor muscle exercises, lifestyle changes, therapeutic adherence, women’s experience

## Abstract

In this study, we aimed to investigate women’s experiences with compliance with prescribed pelvic floor muscle exercises (PFMEs) and lifestyle changes 6–12 months after completing an individual pelvic floor physiotherapy program. This study was targeted to understanding factors affecting adherence to PFMEs and lifestyle changes to deal with pelvic organ prolapse (POP) symptoms. We designed this research as a descriptive qualitative study. We conducted this study from December 2016 to September 2017 in Madrid, Spain. Twenty-six women with symptomatic POP selected using a purposive sampling method participated in six focus groups and three one-to-one semi-structured interviews. Three authors coded and inductively analyzed transcript contents with iterative theme development. A thematic analysis revealed three main themes: (1) symptoms change; (2) PFMEs and lifestyle changes performance; and (3) a health practitioner–patient relationship. Women identified as adherent reported improvement in physical symptoms and emotional and general state as a result of the new knowledge achieved. Fear also promoted compliance with performing PFMEs and adopting lifestyle changes. Likewise, PFMEs preference and routine, integration of PFMEs and lifestyle changes into activities of daily living, support guides, therapeutic alliance, individual supervision, follow-up, and feedback were also identified as adherence facilitators. One of the biggest barriers that we identified was responsibility. Compliance with prescribed PFMEs and lifestyle changes can be improved with effective individual, women-centered, and supervised physiotherapy programs reducing symptoms, including exercises aligned with women’s preferences that are easy to integrate in daily living, promoting knowledge and awareness of their condition, providing written or electronic guidelines, with routine follow-up visits offering both positive feedback and clear and consistent messages, and enhancing therapeutic alliance.

## 1. Introduction

Pelvic organ prolapse (POP) is a common condition affecting mostly postmenopausal women, with a peak age prevalence at 60–69 years [1,2]. The symptoms significantly impair women’s daily activities and quality of life [1,3]. POP involves the descent of one or more perineal organs (uterus, bladder, and/or rectum) from the normal anatomic position into the vagina [4]. Widespread symptoms include feeling vaginal building or pressure in the pelvis, discomfort in the perineum, low back and pelvic pain, which are commonly associated with other urinary, bowel, and sexual symptoms, such as urinary incontinence, fecal incontinence, constipation, and sexual dysfunction that may or may not be related to POP [4,5,6,7]. Moreover, women with POP have reduced pelvic floor muscle (PFM) strength and greater PFM dysfunction, with more severe POP and urinary symptoms [8], and there are weaker PFM involuntary contractions during increases in intra-abdominal pressure such as coughing in women with POP stage I and II than in women without POP [9].

POP can be managed by surgery, conservative management (lifestyle advice, PFM training, and vaginal pessaries), or a combination of these [1,7,10,11]. Conservative treatments are often recommended if the POP is mild or surgery is not indicated. Additionally, surgical treatments are commonly associated with increased risk of postoperative complications and POP recurrence [11,12,13]. According to the latest meta-analysis, pelvic floor muscle exercises (PFMEs) can effectively improve POP symptoms and stage compared to controls [14]. Voluntary PFM contraction may improve support of the pelvic organs as well as their support in the normal anatomic position by contracting PFM before and during any increase in abdominal pressure such as abdominal straining, cough, etc. [14]. PFMEs are more strongly recommended, but their symptom reduction success rates decrease in the medium and long term as adherence to the program deteriorates [15,16]. Although adherence to PFMEs in the short and medium term has been investigated in women with urinary incontinence, women with POP likely also adhere less to PFME programs over time.

To the best of our knowledge, only one study explored adherence to a PFME program in five women with POP in New Zealand with a single data collection method: a one-to-one interview with a single researcher [17].

Thus, in this study, we aimed to investigate the experience with compliance with PFM home exercises and lifestyle changes in a group of women with POP who completed an 8 week supervised pelvic floor physiotherapy program combining PFM physiotherapy, PFM home exercises, and therapeutic education (which included lifestyle changes) in order to understand the factors that can facilitate or inhibit women’s compliance with PFM home exercises and lifestyle changes for improving adherence and person-centered care.

## 2. Materials and Methods

### 2.1. Study Design

We conducted this qualitative research between December 2016 and September 2017 at the Research Unit of the Physiotherapy in Women’s Health Research Group of the University of Alcalá (Madrid, Spain). We selected a descriptive qualitative design [18], and we conducted the study using focus groups and one-to-one interviews [19]. We used focus groups rather than individual interviews mainly because (a) sensitive and personal disclosures are more likely in a focus-group setting, (b) respondents are more likely to be candid when other similar people are present, (c) there is less individual pressure than in an in-depth interview, and (d) the moderator can more easily reintroduce a topic not sufficiently covered than in a one-to-one interview [20]. The study protocol was approved by the Ethics Committee for Clinical Research of the Príncipe de Asturias Hospital (OE10/2010) in Alcalá de Henares, Madrid, Spain. The study reporting followed the Standards for Reporting Qualitative Research (SRQR) guidelines [21], as well as the COREQ checklist [22]. We followed the ethical principles of the Declaration of Helsinki. All participants provided informed written consent.

### 2.2. Participants

Selected using a purposive sampling method [23], we considered 26 women diagnosed by their gynecologist, according to the POP-Quantification Scheme [4], with symptomatic POP, recruited from the Hospital Príncipe de Asturias (Madrid, Spain), and referred to a specialized women’s health unit to receive an individual woman-centeredness program of pelvic floor physiotherapy to manage symptoms of POP for inclusion in the study. Women over 70 years of age, who gave birth within the six months prior to referral, with stage IV POP, with previous surgery for POP, with psychiatric disease, and who were not able to understand Spanish were excluded. Women who completed the physiotherapy program between 6 and 12 months prior to the start of the study were invited to participate.

The pelvic floor physiotherapy program consisted of 16 individual sessions over 8 weeks (two weekly sessions) with a women’s health physiotherapist. The intervention included PFMEs with manual guidance and biofeedback progressing according to the PERFECT scheme from supine to standing and toward functional activities. Women also performed the PFMEs at home, prescribed one to three times per day during the eight-week intervention period. The program also included therapeutic education consisting of instruction with printed and audiovisual materials about the pelvic floor, pelvic floor dysfunctions, the identification of possible precipitating factors, weight loss, constipation, heavy lifting, coughing, high-impact exercise, knack, etc., together with individual strategies for implementing these measures [24]. Upon completion of the physiotherapy program, women were encouraged to continue PFMEs at home (once per day, at least three days per week) and to continue to comply with lifestyle changes.

### 2.3. Data Collection

Women who completed the pelvic floor physiotherapy program 6–12 months ago with an interest in participation were approached by the research team (M.J.Y.-S.) to determine their availability and convey arrangements with the researcher (M.T.-L.) for both conversational-style focus groups and one-to-one interviews. The six focus groups lasted 60 to 70 min each and involved 4, 3, 5, 4, 4, and 3 women, respectively. The three one-to-one interviews lasted 40 to 50 min each. All the focus groups and one-to one interviews were conducted by a facilitator (a physiotherapist experienced in focus groups and one-to one interviewing, M.T.-L.) in private, closed rooms at the Research Unit of the Physiotherapy in Women’s Health Research Group of the University of Alcalá (Madrid, Spain) using a semi-structured approach. In addition, during the focus groups, the facilitator was supported by a qualified pelvic floor physiotherapist (B.N.-B.) who acted as an observer, taking hand-written field notes on nonverbal communications, other observations, etc. We generated the interview questions in the semi-structured guide (Appendix A) with regard to the literature on PFMEs adherence [17,25]. The questions were discussed by the study team: four qualified physiotherapists, two experienced in qualitative research (F.V.-P. and B.S.-S.) and the other two in women’s health (B.N.-B. and M.T.-L.); an experienced gynecologist; and a midwife. All interviews were digitally audio-recorded and later transcribed verbatim by two researchers (B.N.-B. and V.P.-G.), with all women’s names anonymized in the transcripts. We assigned different codes according to the type of interview (focus group or one-to-one interview) and to the women’s intervention order.

### 2.4. Data Analysis

The analysis was conducted by three members of the research team (B.N.-B., F.V.-P., and M.T.-L.). A triangulation process (investigator and data collection) was conducted [26] to ensure rigor in research. We repeatedly read each transcript. Next, in an iterative and consensus process between three researchers, initial codes were generated and described, codes were grouped into higher-order categories, and then the categories were arranged under potential themes. We performed the transcription coding using ATLAS.ti version 6.1 software (Scientific Software Development GMBH, Berlin, Germany).

When the data being collected were repetitive and no new issues were emerging, we considered that data saturation was achieved, so we ceased collecting data [27]. This arose after six focus group interviews and three one-to-one interviews.

## 3. Results

Twenty-six women with symptomatic POP who completed a pelvic floor physiotherapy program 6–12 months ago participated in six focus groups and three one-to one interviews. Two women were Latin American, and the remaining were Caucasian. All women spoke and understood Spanish. The women’s demographics and POP status are shown in Table 1.

**Figure 1:**
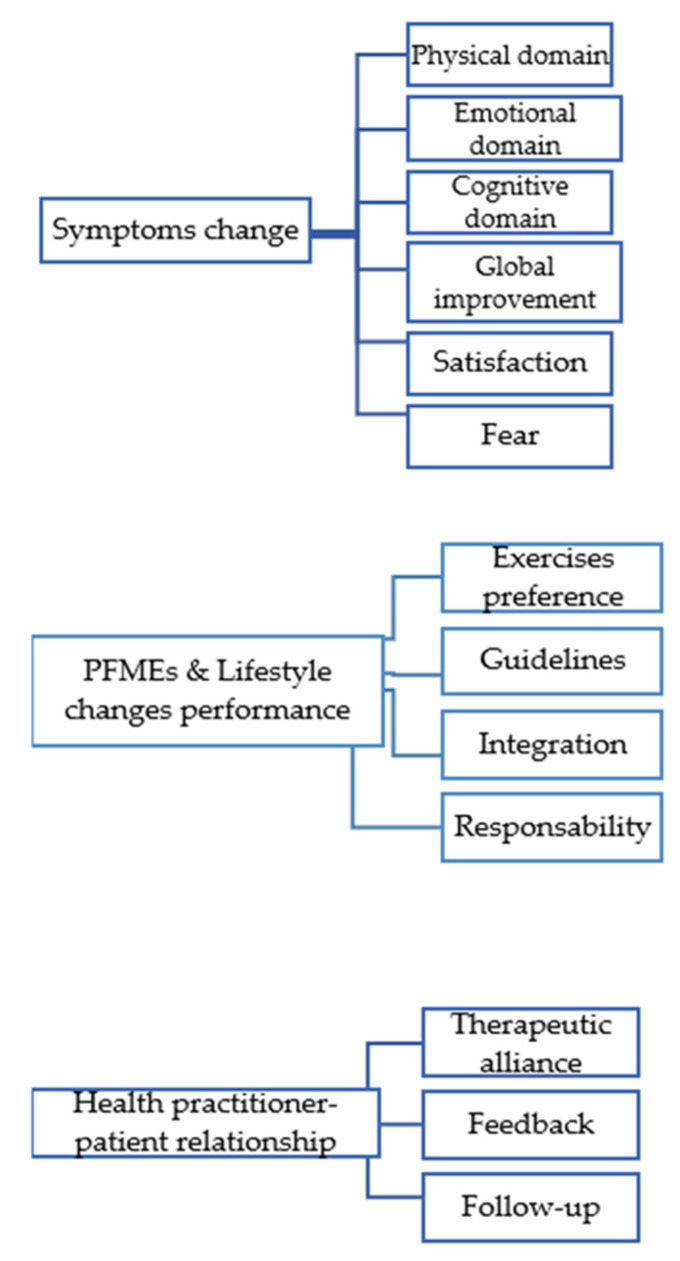
Summary of themes and codes. PFMEs: pelvic floor muscle exercises.

Women’s experiences (facilitators and barriers) in complying with home PFMEs and lifestyle changes were identified in three themes: (1) symptom changes; (2) performance of PFMEs and lifestyle changes; (3) the health practitioner–patient relationship (Figure 1).

### 3.1. Theme 1: Symptom Changes

We identified improvement in physical symptoms as one of the main factors motivating compliance with home PFMEs and lifestyle changes, such as voluntary PFM contractions before and during activities that increase abdominal pressure such as weight bearing, coughing, etc. Women reported that having less of a bulging sensation in the vagina or a reduction in the episodes of urine leakage encouraged them to continue with the exercises.

*I am happy… I no longer leak urine…because before I had frequent leaks. It’s what bothered and I disliked it the most… Now, yes, I feel calm because the exercises that have taught me … I’m fine. (FG3-POP-P5)*

*At the moment I continue to do them [PFMEs and lifestyle changes], mainly contractions before weightbearing and coughing [knack], they have been very good for me, they have been very good for me, I feel much better because before, it seemed that I was walking with a ball there [she points to her vagina] all day, but now I don’t feel anything [an expression of relief spread across her face]. Sometimes when I get constipated, I feel it a bit [the bulge into the vagina], although the position I learned to defecate is better for me, and of course, it is not the same as before coming here to do the exercises and learn those positions to urine, defecate, or contract before coughing, and those things that we learned here...at least I have felt that. (FG2-POP-P4)*

We also found that the fear of worsening symptoms, or feeling the symptoms that they learned to control again, or of surgery as a reminder or trigger to resume the PFMEs and lifestyle changes were facilitating factors.

*As soon as I neglect myself and do the exercises less, or I have an allergy episode that I sneeze many times and I notice heaviness there [pelvis and vagina] or I start again with small leaks [urine leaks], I begin to do the exercise again, to contract [PFM] before sneezing, coughing, etc. Right away I think, I don’t want to go back to be like before … (FG3-POP-P5)*

*I am afraid, afraid that everything will crumble and that is why I force myself to do it [PFMEs and lifestyle changes], because I am afraid. (FG6-POP-P2)*

*Let’s see, my neighbor has been operated and she has not been well. I think about the operation. So, the fear, right? I fear the operation, I know women who have put that surgical mesh on. Very bad. And after three months they have put it back on again, and... I don’t want to go through that. (FG6-POP-P2)*

*I’m afraid that it will fall more, that everything will collapse, and then that will stay dry, and I will get injured and have problems, that is, I am afraid that it will get worse.* *(Int-POP-P3)*

This ability to control their symptoms produced an increase in confidence, security, and satisfaction.

*I was lost, emotionally lost, and everything has improved for me, because I did not know who to turn to and then I already met you one day. Well, my physical condition improved… physically and mentally, I feel more secure. (FG2-POP-P3)*

*I do it [PFMEs and lifestyle changes] to feel better, so that it doesn’t get worse [POP] and improve my quality of life. (Int-POP-P3)*

*You feel satisfied, you know that you can retain it [POP and urine leaks], that you can control it, and that you feel freer. (FG4-POP-P3)*

*… for me it has been a great benefit. I’m very happy”* *(Int-POP-P1)*

They also reported perceiving a global improvement, an improvement in their sexual activity, and even in their quality of life.

*I have learned to control my muscles …. And to also facilitate many things in my body, to know how to control it as well. Sexually it also favors me, that is, it also stimulates many things. And to know my entire body.* *(FG1-POP-P1)*

Women also associated these positive changes with their newly acquired knowledge and shared it with other women. New knowledge about the anatomy and physiology of the pelvic floor, pelvic floor dysfunctions, risk factors, management, how to correctly perform the PFMEs, etc., seemed give them a sense of control, facilitating adherence. The valuable knowledge gained was key to understanding the importance of incorporating PFMEs and lifestyle changes into their routines and living activities to improve their symptoms and thus prevent both POP and other pelvic floor dysfunctions from progressing.

*…everything I have learned here has been surprising for me, and a satisfaction, I had no idea about so many things! Now, I know how important it is for me [PFMEs and lifestyle changes] and I even share it [new knowledge] with my daughters, my friends…* *(Int-POP-P2)*

*Knowing what you can do to improve, why you have to do it, understanding it, is fundamental … Yes, yes, I already contract instinctively, especially when coughing and sneezing, before gaining weight too, I control the stimulating drinks, I no hold myself for so long to go to the bathroom to urine, I defecate in a more adequate posture that helps me not to push… (FG3-POP-P3)*

*And I think that this pelvic floor program should be done for every woman from now on. To adolescents, that they learn everything that we have learned from now on... so that they learn from young, from before getting pregnant to take care of their pelvic floor and that what happens to us does not happen to them. (FG1-POP-P4)*

### 3.2. Theme 2: Performance of PFMEs and Lifestyle Changes

In the theme of performing PFMEs and lifestyle changes, the experience of each woman with PFMEs was included. Women taught to perform the PFMEs felt they were performing them better and that, therefore, they had control (self-efficacy). In addition, the association of PFMEs with some everyday gestures (e.g., voluntary PFM contractions (Knack) before and during activities that increase abdominal pressure such as weight bearing, coughing, etc.,) act as a reminder to perform PFMEs. Therefore, these activities supported compliance.

*But always the same, the one to maintain … I like and am good at. Although, I do PFMEs less than before, really, but everything I learned [lifestyle changes] I have fully integrated, and I no longer have to think about it, I just do it. (FG1-POP-P4)*

*I, for example, the one on “the ladder”, well, the one on “the ladder” and the one “going down and up fast”. I like them and I feel it very well. Also, the one to contract [Knack] before laughing or coughing, I like it a lot because it prevents me from leaks. (FG4-POP-P2)*

*Stand up, especially when I carry weight and sneeze or cough, I feel how it [knack] holds it [POP] inside [into the vagina]. (FG6-POP-P1)*

*That is the one I do best, of course … “the holding” and the knack, drinking, bathroom, etc. [lifestyle changes], that are totally integrated into my life. (Int-POP-P2)*

Most of the women stated that integrating PFMEs and lifestyle changes into their daily life, the possibility of performing PFMEs in any place, and a PFME regular routine (routine of place, routine of time of day) made it easier to continue these changes. Most women claimed to be much more consistent with lifestyle changes, which they considered fully incorporated into their routines.

*...what is easier to do, I see that I can do it [PFMEs] at any time. When I am standing, I am sitting, I am even washing the dishes, I am with the children, because I hold my baby in my arms, and of course, that forces me, because I know that I am going to force myself and that I am going to do it [Knack] at the same time....I take advantage of those moments.* *(FG3-POP-P5)*

*I take advantage of it the most, with the iron, you are standing up, you relax a little and come on, let’s do it [PFMEs]. I really like that. (Int-POP-P2)*

*Yes, I am also…At work doing it [PFMEs] at all times … or waiting for the bus. (Int-POP-P3)*

*… well on Monday, Wednesday and Friday I do these [PFMEs], and on Tuesday and Thursday these others [PFMEs]. Always when I go to bed. (FG6-POP-P3)*

*I have incorporated them [PFMEs] into my gym routine, I do them three times a week that I go to the gym. And I also contract [Knack] when she taught me B. [Pelvic floor program Physiotherapist] during Pilates exercises. (FG4-POP-P4)*

*I always do them [PFMEs] after I shower in the morning, it is already routine and I always do them, just like contracting [Knack] before heavy load, I have already automated it. (Int-POP-P1)*

However, several women reported a lack of criteria regarding the time of day of exercises, repetitions, and progression that made it difficult to perform PFMEs.

*And then when I suddenly remember and say to myself: come on, I’m going to do them at noon, even if it’s on a carpet. I will always win something…. And then at night when I go to bed I say: Uffff! And again I have to do this ….. how many do I have to do, how I continue…? (FG2-POP-P2)*

*And especially when I look bad, I see that I need it and then I do them a few days, oh my God! Why have I stopped it? Pum pum … (FG4-POP-P3)*

In addition, individualized ad hoc guides (written, spoken, or apps) providing reminders regarding PFME types, repetitions, progression, and lifestyle changes were described as support material that acted as a reminder facilitating adherence to PFMEs and lifestyle changes.

*What B. [pelvic floor program Physiotherapist] put in the notebook is what I exactly do, and so I don’t forget. (FG3-POP-P1)*

*I do what B. [pelvic floor program Physiotherapist] taught me, I have it here written down on a paper [written information], and very well. (Int-POP-P1)*

*… I always have a small piece of paper, if I have a doubt...for example: I don’t remember the progression of an exercise, so I look at the paper that I have on the bedside table, and I check the paper. (FG2-POP-P1)*

*Well, I preferred to record it on my mobile phone, and that is how I always carry it with me, and if I don’t remember something, I listen to it quickly, I do it on the subway when I go to work, and it suits me very well. (Int-POP-P3)*

*The apps she [pelvic floor program Physiotherapist] taught me to do the exercises is fantastic, I carry it on my mobile and I do them with the app. I like it. (FG3-POP-P2)*

Finally, the feeling of responsibility and the need to take care of themselves as they take care of others emerged from the sessions, mostly related as a barrier to integrating PFMEs in their daily living. Their roles as caregivers in the family negatively influenced their adherence to PFMEs, since they take care of the family first, and they do not pay enough attention to their self-care.

*It is our responsibility, the disease [POP], but we are not consistent, and we also think before of others, children, family. (FG5-POP-P4)*

*We are not in the habit of spending time with ourselves. Because it is a bad habit and a bad practice of ourselves. So, we always make the mistake thinking “it doesn’t matter”. The last one, us. And we are not interested in ourselves. (FG5-POP-P3)*

*Well, let’s say that our will fails us, the truth is a bit for me that I think I had to be more responsible, and I haven’t done them [PFMEs]. (Int-POP-P1)*

*And we don’t worry about ourselves. Always. And even if we don’t have anything to do, we don’t think about ourselves. (Int-POP-P3)*

### 3.3. Theme 3: Healthcare Practitioner–Patient Relationship

The individualized pelvic floor physiotherapy program including follow-up sessions was identified as essential for performing PFMEs and for compliance with lifestyle changes.

*It is not the same as going to the gynecologist, and they tell you: look, do these exercises that will help you. And then, of course, you say: okay, I do them, but of course, you don’t know if you do it well or do it poorly and of course, here they teach you how to do it. Also, how to urinate or defecate, which before you did not do as well as you should, and other things you do not know. So, it’s different. (FG3-POP-P3)*

*…and B. [pelvic floor program physiotherapist] has been giving me tricks, strategies, helping me to integrate them [PFMEs and lifestyle changes] into my life, to look for the moments … (FG4-POP-P3)*

*While we are here, we do it, we go, at least I do. And I know how to do it and if I do it well is because B. [pelvic floor program physiotherapist] tell me. And then we continue with the reviews. (Int-POP-P2)*

*I thank B. [pelvic floor program physiotherapist] who helped me to improve, because maybe if I did not come here… well, uh… when I also come here for follow-up, I remember to do it, it reinforces me. (Int-POP-P3)*

In addition, the positive feedback of physiotherapists and other health professionals (mainly gynecologists) involved in the therapeutic approach also influenced adherence to PFMEs and lifestyle changes.

*The gynecologist is happy, because he says: we have seen that many people have progressed, and we have seen that I have improved, and POP has been stabilized. (FG3-POP-P3)*

*And it also motivates me that the gynecologist has told me to continue exercising, that I am better and to continue. (FG5-POP-P1)*

The physiotherapists of the program were identified as having a better approach to care than doctors and nurses. The women responded that the aspect of the physiotherapy program that they most valued was their patient, loving, listening, and helpful attitude, reflecting a person-centered attitude (therapeutic alliance).

*….and B. [pelvic floor program physiotherapist] has already taught me with love and patience, and we are learning, because I never knew, and in my country … Well, I am not from here, but now, thank God, with the exercises that B. has taught me, I can. I am very happy and grateful. (FG2-POP-P4)*

*And here [pelvic floor program], I have felt very good, they [pelvic floor program physiotherapists] have treated me very well, with a lot of patience, they have listened to me, they have helped me, and I have learned a lot and I have improved. (FG6-POP-P2)*

*... for the learning we have had, that you can already carry it out to practice it in your daily life with a program that has been made for each of us, and well, if we do not come to the program, we would not have learned all this [PFMEs and lifestyle changes], me at least. You read four things, you know two or three, you know them in your own way, you don’t know if it is well-done as well…. Well, for me it was the best, finding you [pelvic floor program physiotherapists]. (Int-POP-P3)*

## 4. Discussion

In this study, we explored women’s experiences with compliance with home PFMEs and lifestyles changes for POP symptoms and their facilitators and barriers. Our findings showed that most women, over time, do not perform PFMEs regularly (weekly); however, they comply with lifestyle changes. As shown in prior studies, decline in PFME compliance over time is normal [25,31]. Several researchers have investigated the factors that influence adherence to PFMEs, mainly in women with urinary incontinence, although none specifically addressed adherence to lifestyle changes [15,16,32,33,34,35]. Regarding POP, a single qualitative study analyzed adherence to PFME, but it also did not consider adherence to lifestyle changes [17]. So, to the best of our knowledge, this is the first study including factors affecting adherence to lifestyle changes (i.e., strategies for bladder control; contributing factors; knack application to manage everyday pelvic floor challenges; daily PFMEs for at least 15 days after a period of allergy, cold, weight bearing, etc.; performing PFMEs at the end of the sports activities involving weightbearing or impact, in addition to knack; etc.) [24,36,37,38].

Regarding the methodology, we used a descriptive design in order to understand and determine the experience of women who completed a supervised and individualized pelvic floor physiotherapy program including training for the long-term maintenance of PFMEs and lifestyle changes. This approach allowed us to explore women’s experiences with adherence to PFMEs and lifestyle changes for POP from the their perspective and to understand the meanings that the women attach to their behavior [18]. In contrast to the phenomenological study by Hyland et al. [17], a key strength of our descriptive study is the data collection triangulation, combining one-to-one individual interviews and focus groups. The use of investigator triangulation further strengthened this study, resulting in a valid and comprehensive understanding of these phenomena [39].

Our approach produced three themes explaining the factors modifying adherence to PFMEs and lifestyle changes: (1) symptom changes; (2) performing PFMEs and lifestyle changes; and (3) the health practitioner–patient relationship.

### 4.1. Symptom Changes

Changes in symptoms were identified as an important factor modifying adherence. We found that both the improvement in and symptom relapse in those women who stopped performing the PFMEs or relaxed their changes in lifestyle, mainly knack, were powerful triggers of therapeutic adherence. Previous research in women with incontinence reported that women with more frequent urine leaks before and after PFMEs plus an education physiotherapy program were more adherent to PFMEs one year after physiotherapy than women with less frequent urine leaks [15]. Similarly, Abhyankarome et al. reported that women with POP ask for help based on the symptoms experienced. They explored women’s experiences with seeking diagnosis and treatment for POP. They interviewed 22 women receiving POP care through U.K. NHS urogynecology services regarding experiences with seeking diagnosis and treatment for POP and their needs and priorities [40]. Lack of awareness of POP symptoms [40,41] due to mild symptoms resulted in women seeking less help and making less of a sustained effort to perform PFMEs, similar to women with urinary incontinence [15,34,42,43]. However, when women are aware of POP symptoms, when they feel their fear of symptoms worsening, and they feel fear of the condition progressing to the extent that surgery is the only achievable option [40], that is when PFME compliance increases. A wish to avoid surgery as long as possible is a motivator for seeking help and for performing PFMEs [40].

These symptoms can affect women’s self-esteem, body image, and quality of life [40] and can have social, psychological, and sexual impacts. The more the symptoms progress, the more they affect quality of life, social well-being, and sexual health [44,45,46]. Ghetti et al. found, in their qualitative study describing the emotional burden experienced by forty-four women seeking treatment for POP, that women’s psychological well-being is intimately related with their POP symptoms [47]. Salovey et al. reported that physical health and emotional states influence one another. Positive emotional states, such as the satisfaction expressed by the women in this study, and their empowerment regarding the control of their symptoms may promote healthy perceptions, beliefs, and physical well-being [48]. Women also linked satisfaction and sense of control to embodied knowledge. Women stated that gaps in their pelvic health knowledge have been addressed. Pintos-Díaz et al., in a qualitative study exploring the reasons Spanish women with urinary incontinence sought help, stated that women’ have complaints about the overall lack of information. They also found that the knowledge of many women about pelvic floor and pelvic floor dysfunctions was based on beliefs or myths [43]. Pelvic health education is effective in dealing with myths, false beliefs, and misinformation about the pelvic floor and pelvic floor dysfunction [43]. Moreover, all women reported using their new knowledge, mainly lifestyle changes, and PFMEs. They even shared this information with others. Adherence was linked with knowledge. The more knowledgeable and insightful the women were about their pelvic floor and pelvic health, and the more they experienced an effect on their symptoms, the more they adhered to PFMEs [49]. These findings agree with those of other studies conducted in women with urinary incontinence [15,50,51].

This theme did not emerge the study by Hyland et al. [17]. This could be due to the clinical characteristics of the women in each study. Although women were homogeneous regarding the stage of POP (II and III) and they could also have been homogeneous in relation to the POP symptoms, whether the five women interviewed by Hyland et al. suffered from other pelvic floor dysfunctions is unknown. In our study, women also reported urinary incontinence (76.9%) and anal incontinence (46.4%) symptoms. POP often co-exists with other pelvic floor dysfunctions, mainly those involving urinary incontinence [5,12,52].

### 4.2. Performing PFMEs and Lifestyle Changes

Compliance with PFMEs and lifestyle changes was associated with regular routines and their integration into their daily lives, PFME movability, and PFME preference regarding control and self-efficacy. Most women performed the PFMEs, which allowed them to have more control and a greater sense of performing PFMEs effectively, which could be related to self-efficacy with a PFME-specific task. A woman’s belief in her own ability to perform PFMEs seems to influence adherence to PFME as well as to predict the intention to adhere to PFMEs [15,36,53,54]. These findings agree with those of prior studies in women with urinary incontinence [34] as well as with the findings reported by Hyland et al. in women with POP symptoms [17].

Women also valued the individualized ad hoc guidelines because they explained PFME types, repetitions, progression, and some lifestyle changes. This support material acted as a reminder that facilitated adherence to PFMEs and lifestyle changes. Several studies have evaluated strategies to improve adherence to PFMEs [15,55,56,57] in women with urinary incontinence. Some researchers tested the efficacy of electronic reminders or exercise diaries, finding that these are effective in increasing adherence to PFMEs in an unsupervised approach [55,56,57]. As barriers to seeking treatment, Abhyankarome et al. reported that women with POP expressed forgetting to perform the exercises after some time. To recall the exercises, some of them used telephone apps or alarms as reminders and some paired the exercises with daily activities [40].

Women identified the largest barrier to PFME adherence as the feeling of responsibility and the need to take care of others. In the current study, not only did women prioritize others over themselves, but they also expressed their poor commitment to their own healthcare. This may be due to the role of women in society as family planners and caregivers [58,59], probably because of their feeling of moral and affective obligation [60,61]. Despite the social changes over the last decades and the increased involvement of men in caring for the family, women continue to be the main caregivers, at least in Spanish society [62]. These findings agree with those of Alewijse [15] and Hyland [17].

In studies on PFME adherence, adherence is associated with performing PFMEs, seldom with bladder training or with the functional use of pelvic floor muscles in activities of daily living. In our study, women stated that they were more compliant with the functional use of pelvic floor muscles in their day-to-day activities (i.e., knack) than with PFMEs. Moreover, the functional use of the pelvic floor muscles acts as a reminder to perform PFMEs, and therefore supports compliance. This is probably because knack is an effective tool in reducing urine loss, as well as a valid tool for reducing bladder neck movement, which can also minimize POP symptoms [63,64]. To the best of our knowledge, this is the first study exploring adherence to lifestyle changes (including functional use of the pelvic floor muscles) in women with POP symptoms.

### 4.3. Health Practitioner–Patient Relationship

Women in our study highlighted the importance of their relationship with healthcare professionals, mainly with the gynecologist and with physiotherapists. This could because these women underwent a previous pelvic floor physiotherapy program, and in this eight-week program, the physiotherapists had more opportunities to explain the program and have discussions with the women. The most valued aspect, as in Abhyankarome et al., was the physiotherapists’ person-centered attitude. This issue has not emerged in other studies on adherence to PFMEs. This could be because those studies were less supervised or the interventions were unsupervised. The therapeutic relationship or therapeutic alliance seems to improve adherence, satisfaction, and quality of life [65].

This therapeutic alliance may have also been reinforced by the follow-ups and the univocal positive feedback from all of the health professionals involved in the diagnosis and treatment of the women in the study. The follow-up and feedback were described as a demand from women with symptoms of POP, expressing “a strong need for longer-term monitoring and periodic follow-up of prolapse symptoms following the appropriate training in PFME” [sic] [40]. These follow-ups and univocal positive feedback may have simultaneously reinforced self-efficacy [35].

Our findings of the experiences with long-term PFME adherence confirm those reported in the literature on women with urinary incontinence [15,16,25,32,33,34] and with POP symptoms [17]. Our findings also provide new evidence of the experiences of women with POP symptoms and adherence to lifestyle changes.

### 4.4. Limitations

The qualitative approach employed in this study also has some limitations. By nature, this approach prevents the generalization of the findings to further ethnically diverse groups. Although the purpose of qualitative research is not to generalize, our results can be extended mainly to Spanish-culture Caucasian women (although two Latin American women also participated) who have previously attended an individualized and supervised woman-centered pelvic floor physiotherapy program in an urban and outpatient academic and research women’s health physiotherapy unit. Therefore, our findings cannot be extended to women with POP symptoms who have not visited a women’s health clinic or specialist.

Although the interviewer (facilitator, M.T.-L.) was an independent researcher to the pelvic floor physiotherapy program that all women previously completed, the observer (B.N.-B.) in the focus groups interviews was one of the pelvic floor physiotherapy program physiotherapists. Therefore, women may also have said what they thought the physiotherapist would have wanted to hear. Nevertheless, the fact that the women and one of the researchers had a relationship may have contributed to a safe, confidential, and conducive environment for women to talk about their intimate stories.

## 5. Conclusions

To conclude, long-term compliance with prescribed PFMEs and lifestyle changes can improve with effective, individual, women-centered, and supervised pelvic floor physiotherapy programs, thereby reducing symptoms. The program should include exercises agreeing with women’s preferences that are easy to integrate into their activities of daily living, promoting knowledge and awareness of their condition, providing written or electronic guidelines adapted to the specific needs of women, with routine follow-up visits offering both positive feedback and clear and consistent messages from all of the healthcare professionals involved, and enhancing the therapeutic alliance.

An increased understanding of the facilitators of and barriers to performing PFMEs and lifestyle changes may help providers better understand women’s needs and provide individualized care.

## Figures and Tables

**Table 1:** Women’s demographics and prolapse status.

Parameter	Value
Age (years), X(SD)	57(9)
BMI (kg/m^2^), X(SD)	25.1(4.7)
Menopause, *n* (%)	26 (92.8%)
Type of prolapse, *n* (%)	
Cystocele	20 (71.4%)
Hysterocele	12 (42.8%)
Rectocele	5 (5.3%)
Stage of prolapse, *n* (%)	
1	0 (0%)
2	21 (75%)
3	7 (25%)
4	0 (0%)
Other PFD	
Urinary incontinence	20 (76.9%)
SUI	11 (55%)
UUI	4 (2%)
MUI	12 (60%)
Anal incontinence	13 (46.4%)
Flat	12 (92.3%)
Flat & FI	1 (7.7%)
Time between physiotherapy program and interview (months), X (SD)	10.1 (1.4)
Pre-Post physiotherapy program changes P-QoL score (points), X (SD)	22.3 (5.6) *
PFDI-20 score (points), X (SD)	−28.4 (−19.3) **
PFM strength (cmH_2_O), X (SD)	9.78 (2.48) ***

BMI: Body mass index; PFD: Pelvic floor dysfunction; SUI: stress urinary incontinence; UUI: urgency urinary incontinence; MUI: mixed urinary incontinence; FI: Fecal incontinence; P-QoL: Prolapse Quality of Life Questionnaire (* an improvement of 14.5 points is considered clinically relevant [28]); PFDI-20: Pelvic Floor Distress Inventory Short Form (** an improvement of 13.5 points is considered clinically relevant [29]); *** an improvement of 9 cmH_2_O is considered clinically relevant [30]; PFM: Pelvic floor muscles; X (SD): Mean (Standard Deviation).

## Data Availability

Data are held securely by the research team and may be available upon reasonable request and with relevant approvals in place.

## Appendix A

**Interview Guide**
What do you think the pelvic floor muscle exercises are for? What do you think are its effects?
What do you think the lifestyle changes are for? What do you think are its effects? Can you tell me about the kind of things you do when you do pelvic floor muscle exercises? Where are you? What time of day?
What exercises do you practice the most? Why?
What exercises do you practice the least? Why?
What do you think makes it easier to do the exercises?
What do you think makes it difficult to do the exercises?
Tell me about how your exercises fit into a typical day Tell me about how your exercises fit into a typical week How do you go about fitting these exercises in? Can you tell me about your lifestyle changes? What have you adopted? Where are you? What time of day? What do you think makes it easier lifestyle changes? What do you think makes it difficult lifestyle changes? Tell me about how your lifestyle changes into a typical day Tell me about how your lifestyle changes into a typical week How do you go about fitting these lifestyle changes in? What things get in the way of you doing your exercises? And making your lifestyle changes? How do you cope with these “barriers”? What have you found useful to keep you going with exercising? What have you found useful to keep you going with lifestyle changes?
